# Placental Findings in Lysosomal Storage Disease Diagnosis: A Case Report of Galactosialidosis

**DOI:** 10.1155/2020/8181056

**Published:** 2020-05-30

**Authors:** Sasha Libbrecht, Francois Eyskens, Sabine Declercq, Cecile Colpaert

**Affiliations:** ^1^Department of Pathology, UZ Gent, De Pintelaan 185, 9000 Gent, Belgium; ^2^Centre for Metabolic Diseases, University Hospital Antwerp, UZA, Wilrijkstraat 10, 2650 Edegem, Belgium; ^3^Department of Pathology, ZNA, Lindendreef 1, 2020 Antwerpen, Belgium; ^4^Department of Pathology, UZA, Wilrijkstraat 10, 2650 Edegem, Belgium

## Abstract

*Introduction*. Lysosomal storage disorders (LSDs) are rare diseases with more than 50 different entities described today. The spectrum of phenotypes varies from severe to lethal and early-onset disease to mild and late onset. Recognition of the clinical signs and diagnostic workup is challenging and requires expertise. Diagnosis relies on finding abnormal metabolites in urine and serum followed by further enzymatic or molecular analysis. Routine histological examination of the foetal and placental tissues frequently shows vacuolisation, providing a readily available important clue to the diagnosis. *Case Report*. A third child of consanguineal parents showed several dysmorphic features and a complicated neonatal period with eventual demise in the early postneonatal period due to respiratory failure. An LSD was suspected based on clinical presentation, urine metabolite excretion, skeletal radiograph, and vacuolisation in lymphocytes and placental tissues on, respectively, blood smear and routine histological examination. Homozygosity mapping favoured galactosialidosis. The diagnosis was confirmed by massive parallel sequencing, revealing a single nucleotide variation in the CTSA gene (c.265A>C, p.Ser89Arg). *Discussion*. Histological placental examination may be either the first clue or complimentary evidence in recognizing LSDs. It is important to recognize these clues as it may prompt further investigation and facilitate earlier recognition of the disease.

## 1. Introduction

Lysosomal storage disorders (LSDs) are a large and heterogenous group of congenital metabolic diseases. This group consists of more than 50 different entities, each showing lysosomal accumulation of various undegraded metabolites in a vast array of body tissues. The buildup of these metabolites results in tissue malfunction, leading to multisystemic disease. The onset of symptoms is heterogenous, with some diseases having an early and severe phenotype while others appear to be mild, with symptoms appearing only later in life. The different lysosomal storage disorders are rare, ranging from 1 per 57,000 live births for Gaucher disease to 1 per 4.2 million live births for sialidosis. Although worldwide epidemiological data on LSDs is limited, as a group, the incidence is estimated at around 1 : 5,000–1 : 8,000 [1].

The majority of LSDs are inherited in an autosomal recessive fashion, while a couple (Fabry disease, Hunter syndrome, and Danon disease) are X-linked diseases. The cause of metabolite accumulation is usually due to a defect in specific lysosomal enzymes or, less frequently, in nonenzymatic lysosomal proteins or proteins involved in lysosomal biogenesis. In the past, these diseases were classified based on their accumulated substrate(s). More recently, classification based on the specific molecular defect is favoured, mainly due to a growing understanding of the molecular basis of LSDs. This classification helps to elucidate the mechanisms of lysosomal storage malfunction and in some cases subdivides some LSDs further by their distinctive faulty mechanism (e.g., sphingolipidosis group that can owe their undegraded sphingolipid accumulation to a defective enzyme or an activator protein defect). Filocamo and Morrone discuss the classification, molecular basis, and laboratory findings of LSDs in more detail [2].

The recognition of clinical features suggesting LSD requires expertise, as the symptoms and other findings are often unspecific and can be caused by defects in different metabolic pathways or environmental factors. Even if an LSD is suspected, the definitive diagnosis can still be challenging as the diagnostic tests differ for various groups of lysosomal storage diseases. Preliminary tests on urine, serum, or blood, in most cases identifying increased undegraded metabolites, can assist in the diagnosis and in choosing the appropriate enzymatic or molecular analysis.

Histologically, the accumulation of metabolites results in vacuolisation of different tissues from the affected individual. In severe early-onset LSDs, resulting in intrauterine foetal or neonatal death, body tissue may not always be available for further examination. Similar alterations can also be found on histological examination of the placenta. This may help steer the diagnosis towards LSDs as a group or sometimes even suggest a specific LSD and thus facilitate or prompt further investigations [3].

Individuals with galactosialidosis, the focus of our case report, show a defective activity of two enzymes, b-galactosidase (*β*-GAL) and neuraminidase 1 (NEU1). This is caused by mutations in the CTSA gene, encoding lysosomal protective protein cathepsin A (PPCA). PPCA forms a complex with *β*-GAL and NEU1 and carries out a protective function. The lack of PPCA results in a combined *β*-GAL/NEU1 deficiency.

We report a case of galactosialidosis in which vacuolisation in placental tissues and lymphocytes, on, respectively, routine histological examination and blood smear, instigated further investigations which led to a definitive diagnosis.

## 2. Case Report

A third child of consanguineal parents showed multiple dysmorphic features on routine prenatal sonography. The foetus displayed facial abnormalities (retrognathia, protruding ears), deformed feet (sandal gap, club foot), polyhydramnios, subcutaneous oedema, hydrocele, and ascites (hydrops fetalis). Further prenatal diagnostic tests were refused by the parents for religious reasons. The two other children of the couple were in good health and showed no congenital abnormalities.

At 37 weeks, 3 days from LMP, a baby boy was born. In addition to the prenatal findings, generalized petechia (mainly on the face) and a simian crease were observed. The baby showed a high birthweight for gestational age (3,680 g, >p90). Due to tachypnoea, continuous positive airway pressure was given over a short period of time, followed by high airflow via the nasal cannula. A week after birth, progressive respiratory distress was seen with diffuse lung opacities on chest radiograph. Further deterioration and a need for intubation followed. An infectious agent was suspected although cultures remained negative. The child died, just a little more than a month after birth, due to respiratory failure. Autopsy was refused by the parents.

During hospitalization, bloodwork showed anaemia, thrombopenia, hypoalbuminemia, elevated liver enzymes (alkaline phosphatase and *γ*-GT), and vacuolated lymphocytes. Urine analysis showed proteinuria and oligosacchariduria. Abdominal ultrasound demonstrated an enlarged liver and spleen. The babygram findings were notable for osteopenia, rough trabecular aspect of the bones, and metaphyseal cupping and fraying.

**Figure 1:**
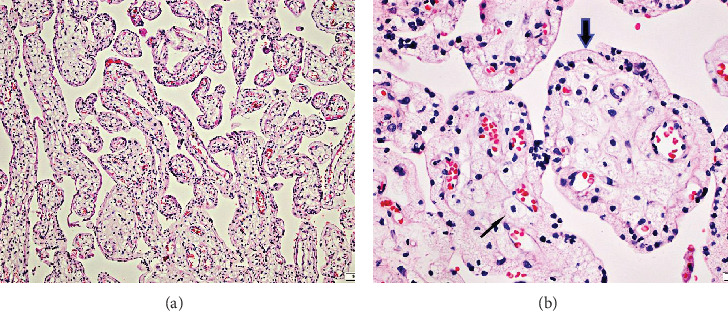
H&E section of the 3rd trimester placenta. An overview (a, original magnification ×4) shows pale enlarged villi with a distended pale trophoblast layer and foamy appearance of the villous stromal cells. On higher magnification (b, original magnification ×20), coarse vacuolisation in syncytiotrophoblast (thick arrow) and stromal cells (thin arrow) is observed.

The placenta was large and heavy (p95). Routine histological examination revealed enlarged hydropic pale villi lined by vacuolated syncytiotrophoblast and a foamy, vacuolated appearance of villous stromal cells (Figures 1(a) and 1(b)).

Clinical findings suggested a severe, early-onset metabolic disorder. Because of the blood smear findings (vacuolated lymphocytes), urinalysis (oligosacchariduria), radiologic skeletal findings, and placental examination, a lysosomal storage disease was suspected. The final differential diagnosis for this clinical presentation, together with the first available laboratory tests, suggested either infantile sialidosis or galactosialidosis. Homozygosity mapping favoured galactosialidosis. This diagnosis was confirmed on a whole blood sample from the infant by massive parallel sequencing with an LSD gene panel, finding a homozygous mutation in the CTSA gene (c.265A>C, p.Ser89Arg).

The parents came in for genetic counselling and were informed about the odds of recurrence (25%) in a future pregnancy. As both parents wished to have another child, different precautionary measures such as chorionic villus sampling and preimplantation diagnosis by way of in vitro fertilization were discussed.

## 3. Discussion

Histological placental examination is a fast and low-cost investigation that can be carried out in all surgical pathology laboratories. It may prove to be a great tool in the workup of complicated pregnancies, including but not limited to intrauterine death, perinatal death, intrauterine growth restriction, and hydrops fetalis [4].

We present a case of hydrops fetalis where routine placental examination showed a heavy and large placenta for gestational age. On histological examination, vacuolisation in the syncytiotrophoblast and villous stromal cells was evident (Figures 1(a) and 1(b)). Coinciding finding of vacuolated lymphocytes on blood smear motivated further investigations that led to the diagnosis of galactosialidosis. Since autopsy was refused by the parents, foetal tissues were not available for examination.

Our findings of placental histology in galactosialidosis were similar to ones previously reported in the literature [5].

Many LSDs may lead to nonimmune hydrops fetalis (NIH). In a review by Gimovsky et al., LSDs accounted for up to 5.2% of NIH and up to 17.4% if only idiopathic NIH was considered [6]. Often, these cases are only recognized as LSDs after recurrent pregnancies complicated by hydrops fetalis [7, 8]. Any clues pointing towards metabolite storage abnormalities may therefore be very helpful in achieving an earlier diagnosis.

Additionally, paying attention to the distribution of inclusions or vacuoles in different placental cells as well as the type of vacuolisation may also be of help in further steering of the diagnostic approach. Different LSDs seem to have distinct cell populations in the placenta that are most affected. For example, while galactosialidosis shows coarse vacuolisation in the villous stroma and syncytiotrophoblast, other LSDs only show vacuolisation in the stromal cells without affecting the trophoblast (mucopolysaccharidosis), vacuoles in intermediate trophoblast (mucolipidosis type II), and vacuolisation in Hofbauer cells (*β*-glucuronidase deficiency). However, much overlap is seen. Benirschke et al.'s *Pathology of the Human Placenta* provides a summary on placental histology in LSDs [3].

Although the placenta is often enlarged or hydropic in cases of LSD, vacuolisation or inclusions are not always evident [3]. A thorough examination of the placenta, especially in cases with NIH, is therefore warranted.
